# Postoperative discomfort of dental rehabilitation under general anesthesia

**Published:** 2014

**Authors:** Kenan Cantekin, Mustafa Denizhan Yildirim, Ebru Delikan, Seçil Çetin

**Affiliations:** 1 Kenan Cantekin, DDS, PhD, Assistant Professor, Department of Pediatric Dentistry, Faculty of Dentistry, Erciyes University, Kayseri, Turkey.; 2 Mustafa Denizhan Yildirim, MD, Assistant Professor, Department of Pediatric Dentistry, Faculty of Dentistry, Erciyes University, Kayseri, Turkey.; 3 Ebru Delikan, DDS, Research Assistant, Department of Pediatric Dentistry, Faculty of Dentistry, Erciyes University, Kayseri, Turkey.; 4 Seçil Çetin, DDS, Research Assistant, Department of Pediatric Dentistry, Faculty of Dentistry, Erciyes University, Kayseri, Turkey.

**Keywords:** General anesthesia, Dental care for children, Dental caries, Extraction

## Abstract

***Objective:*** To find out postoperative discomfort in children undergoing dental rehabilitation under general anesthesia (DRGA).

***Methods: ***This study involved 78 (4 to 10 year-old) healthy patients who were scheduled for DRGA and were needed extensive dental treatment because of severe caries, and showed high dental fear and/or behavioral management problems. The children had to be fit for DRGA administration by fulfilling the American Society of Anesthesiologists physical status I or II and no associated mental health or communication problems. Data were collected by structured interview either face to face (immediately post operation) or using a telephone (post operation after discharge). One of the study’s investigators recorded all data related to the immediate postoperative period during the child’s stay in the post-anesthesia care unit (PACU). The questionnaire consisted of questions related to postoperative problems experienced by the patient in the period after their day-stay attendance. The questionnaire, consisting of questions regarding and generally related to the child’s activities. In addition, pain was assessed using the face, legs, activity, cry, consolability (FLACC) scale.

***Results: ***The prevalence of postoperative problems was 46 out of 78 (59%). The mean FLACC score was 1.8 (SD=2.1). Some of the patients having more than one reported problem. Forty-one percent of the children showed nasal discomfort (*P<*0.01). Thirty-three percent and 43% of the children experienced throat or mouth discomfort. The most common experienced postoperative symptom after DRGA was bleeding. Nasal bleeding, however, was an uncommon complication and did not cause serious morbidity or mortality in children intubated nasotracheally. In addition, postoperative discomfort was related to number of the extractions. Children who had 4 or more extractions were more likely to experience pain. Findings associated with other bodily functions were assessed. Nausea and vomiting were reported in 20.5% of children. Twenty-six children (18%) had a fever. Thirty-nine (50.0%) parents reported that their children had problems eating.

***Conclusion:*** Post-operative discomfort was more with 4 or more extraction done under DRGA and that nasal bleeding was noted a uncommon post-operative symptom.

## INTRODUCTION

In developing countries, dental caries remains one of the most prevalent health problems in children. Comprehensive dental rehabilitation under general anesthesia (DRGA) is a treatment option for children who require extensive dental treatment, exhibit acute situational anxiety and emotional or cognitive immaturity, or are medically compromised.^1^ There are several advantages of DRGA, including safety, efficiency, convenience, and high-quality restorative and preventive (e.g., fissure sealing) dental treatment.^2^

 Dental practitioners usually have limited contact with parents after DRGA. It might be anticipated that the route of intubation could result in discomfort in and around the nose and throat areas.

There have been very few reports published on complications after DRGA involving nasal intubation. The purpose of this study was to investigate postoperative discomfort in children undergoing DRGA via oral and nasal intubation in the first 24 hours after discharge.

## METHODS

The study was approved by the Ethics Review Board of the Medical Faculty of Erciyes University (Turkey). The participants were a consecutive clinical convenience sample of the parents/caregivers of children receiving DRGA at Gülşah and Mert Tatar Mouth, Teeth and Jaw Surgery Hospital of Erciyes University, Turkey. DRGA was considered necessary if the child was 4–10 years old, needed extensive dental treatment because of severe caries, and showed high dental fear and/or behavioral management problems. The children had to be fit for DRGA administration by fulfilling the American Society of Anesthesiologists physical status I or II and no associated mental health or communication problems.

One hundred and twelve children were initially screened for consideration of DRGA, but only 78 children were included in the study. The reasons for exclusion are given in Table-I. Carious teeth of the children were restored by traditional restorative treatment (e.g., composite, compomer, stainless steel crown, and pulp therapy), or extracted if unrestorable.

Information regarding the study was given to potential participants in the form of a written covering letter / information sheet, with further verbal information provided by the same pediatric dentist (KC) if necessary. Written consent was obtained.

Upon arrival in the operating room, standard monitors (including electrocardiogram, pulse oximeter, and blood pressure cuff) were applied to the children. Anesthesia was induced with 8% sevoflurane in oxygen, at a total fresh gas flow of 6 lt / min, via a pediatric circle circuit. When the response to verbal command was lost, intravenous access was established and nasal or oral intubation was facilitated with i.v rocuronium 0.6 mg / kg .Ventilation continued with 2% inspired sevoflurane in a 50:50% mixture of oxygen/air. At the end of the dental procedure, residual neuromuscular blockade was antagonized with atropine 0.02 mg/kg IV and neostigmine 0.05 mg/kg IV. The trachea was extubated when the patient made purposeful movements and had a regular respiratory pattern.

***Data Collection: ***Data were collected by structured interview either face to face (immediately post operation) or using a telephone (post operation after discharge). One of the study’s investigators recorded all data related to the immediate postoperative period during the child’s stay in the post-anesthesia care unit (PACU). The questionnaire consisted of questions related to postoperative problems experienced by the patient in the period after their day-stay attendance. The questionnaire, consisting of questions regarding and generally related to the child’s activities, is shown in Table-II. In addition, pain was assessed using the face, legs, activity, cry, consolability (FLACC) scale (Table-III).^3^

***Pain Assessment: ***The FLACC pain assessment scale was the primary instrument used by PACU nurses and parents to assess the prevalence and intensity of the child’s pain in this study. The FLACC pain assessment scale incorporates five categories of pain behaviors: (1) facial expression; (2) leg movement; (3) activity; (4) crying; and (5) consolability. Each category is scored from 0 to 2, resulting in a total score between 0 and 10 (Table-III).

***Statistical Analyses: ***Data management and analyses were conducted using SPSS version 16.0 (SPSS, Inc., Chicago, Ill., USA). Mean age and gender distribution across groups were calculated. Potential risk factors for postoperative complications were assessed using bivariate analysis and the chi-square test. A *P*-value < 0.05 was accepted as statistically significant. After identifying significant variables by bivariate analysis, these were entered into multiple logistic regression models to examine each variable, while controlling for all other confounding factors.

## RESULTS

***Socio-Demographic and Self Care Data: ***The sample consisted of 41 boys (52.5%) and 37 girls (47.5%), with mean ages of 6.12±1.73 years. The mean monthly family income was US $1,286. The mean DMFT scores were 11.33±2.60.

***Dental Treatments and Duration of Procedure: ***Extractions were provided for 66 (86.6%) of the children. Restorations were provided for each of 78 children (100%) with a mean of 7.24); 16 children (11.5%) had restorative treatment without any extractions being done; and no children (0%) had extractions without any restorative work being provided.

Of the cases, 18% could be deemed as “short,” lasting up to 30 minutes. The majority, 62% were intermediate, lasting up to an hour, and 20% were long, lasting over an hour. Five children had procedures lasting over 90 min. Mean anesthetic time was 58±28.3 min and the mean recovery time was 16±8.1 min.

***Postoperative discomfort: ***The prevalence of postoperative problems was 46 out of 78 (59%) in the children, with some of the patients having more than one reported problem. Forty-one percent of the children showed nasal discomfort (*P<*0.01). Thirty-three percent and 43% of the children experienced throat or mouth discomfort.

***Postoperative Pain: ***Fifty-one percent of children had a FLACC score greater than 0 at the time of discharge; however, 27% had a score of 3 or more. The mean FLACC scores in the children was 1.8 (SD=2.1).

Observed patterns were confirmed in both the bivariate and multivariate analysis. Those children who had extractions as part of their dental rehabilitation were more likely to experience pain, as reported by FLACC scores in the PACU (*P*=0.01; Table-IV). There was no relationship between reported FLACC scores and gender (*P*=0.19).

***Postoperative Bleeding: ***Concerning findings associated with bleeding, 59.0% of nasal intubated children were reported to exhibit this complication. Of these, 79.3% of the children witnessed bleeding from the mouth, whereas 12.8% of children had blood from the nose.

Findings associated with other bodily functions were assessed. Nausea and vomiting were reported in 20.5% of children. Twenty-six children (18%) had a fever. Thirty-nine (50.0%) parents reported that their children had problems eating. 

## DISCUSSION

Turkish children are mostly referred for DRGA when they have a significant number of decayed teeth and cannot be treated in standard clinical conditions. At the population level, DRGA is commonly needed in Turkey, where the majority of children have decayed teeth; 69.8% of five-year-old children had caries in 2011.^4^ While most children are able to undergo dental treatment in the conventional setting, some patients are too young or fail to respond to the usual behavior management techniques.^5^^,^^6^ The benefits of children’s dental care under general anesthesia are full-mouth rehabilitation in a single appointment and instant pain relief. In addition, such treatment requires little or no cooperation on the part of young children.^7^^,^^8^ Our hospital uses DRGA only as a last resort, when treatment cannot be conducted by other means, in line with the current guidelines on the use of GA in dentistry.^9^

**Table-I:** Exclusion criteria for present study

***General reasons***	***Nasal intubation***
Not ultimately willing to participate (withdrawal)	Gastroesophageal reflux
Older and younger than the study’s age range	History of latex allergy
Inability to connect with the caregivers/parents of the participants.	History of allergy for anesthetic agents
Failure to return for follow-up.	Abnormal coagulation status
	Recurrent epistaxis
	Nasal polyposis

**Table-II:** Post-operative complications of naso-trachealy intubated children

***Complications***	***Nasal N=78*** ***(n/%)***
Problems in eating	42 (53.8)
Nose discomfort	32 (41.0)
Throat discomfort	26 (33.3)
Mouth discomfort	34 (43.6)
Dental pain	28 (35.9)
Bleeding	46 (59.0)
Epistaxis	10 (12.8)
Pain medication	20 (25.6)
Diarrhea	4 (5.1)
Constipation	14 (17.9)
Nausea-vomiting	16 (20.5)
Fever	12 (15.4)

**Table-III:** The face, legs, activity, cry, consolability (FLACC) pain assessment scale

**Parameters**	**Scores**		
	**0**	**1**	**2**
**Face**	No particular expression or smile	Occasional grimace or frown, withdrawn, disinterested	Frequent to constant quivering chin, clenched jaw
**Legs**	Normal position or relaxed	Uneasy, restless, tense	Kicking or legs drawn up
**Activity**	Lying quietly, normal position, moves easily	Squirming, shifting back and forth, tense	Arched, rigid, or jerking
**Cry**	No cry (awake or asleep)	Moans or whimpers, occasional complaint	Crying steadily, screams or sobs, frequent complaints
**Consolability**	Content, relaxed	Reassured by occasional touching, hugging, or being talked to, distractible	Difficult to console or comfort

**Table-IV:** Extractions and FLACC scores in each group

***Extraction (N)***	***FLACC scores***		
	***N***	***Mean±SD***	***CI(95%)***
0	22	0.8±0.9^a^	0.4-0.6
1-3	37	1.6±1.7^b^	0.9-1.4
≥4	29	2.3±2.6^c^	1.1-2.1

KC: Designed the Protocol and prepared the final manuscript,MDY: Performed general anesthesia procedures,

ED: was involved in data collection, SC: was involved in data collection.

It seems likely that nasotracheal intubation poses few problems in terms of post-anesthetic discomfort in most children; our data suggest that a small proportion of patients may complain of some nose discomfort. We observed that 29% of the children suffered from sore throat in PACU. Similarly, Needleman et al.^10^ and Holt et al.^11^ reported identical prevalence rates of 27% for children with sore throat postoperatively. Most subjects in our study were intubated atraumatically. As expected, all of the seven subjects who had traumatic intubations had sore throats in the PACU.

In the present study, postoperative pain was only experienced by 29.7% of the patients. However, Atan et al.^12^ evaluated all the postoperative outcomes and reported that pain lasted the longest. Pain was also a common finding in the majority of previous studies investigating postoperative pain in children after receiving DRGA, the incidence of which ranged between 36% and 95%.^10^^-^^15^ In accordance with our results, three studies reported pain to be an uncommon occurrence (0–8%).^14^^,^^16^^,^^17^ It is important to note when comparing these studies that there is great variability among the study designs, such as the method and time(s) of pain assessment, the age and medical status of the children, and the quantity and types of procedures performed. Another possible explanation of the difference from the aforementioned studies may be that majority of the dental practitioners did not administer local anesthesia before extractions. In our study, intraoperative local anesthesia was routinely used; this likely diminished the pain in the postoperative period.

The present study demonstrated a relationship between extraction and FLACC scores. Extractions caused more postoperative pain, and children who had more than four extractions were more likely to report pain. This finding agrees with several previously published studies. Needleman et al.^10^ suggested that children with extractions as the most invasive dental procedure were 7-fold more likely to report pain during the postoperative period. Noble et al.^18^ found that the greater the number of teeth extracted, the greater amount of distress reported. Similarly, Atan et al. reported that the odds of experiencing pain were elevated with increasing numbers of surgical procedures.^12^ Therefore, in DRGA including extractions, it is important to instruct parents to give analgesics regularly on the first postoperative day.

In the present study, we witnessed nasal hemorrhage for only 12.8% of nasally intubated children. However, of all of the children who present with nasal bleeding, this constituted minor bleeding, characterized as a small amount of blood tinged mucous in the oropharynx post intubation. None of the patients in this series had any complications that resulted in serious morbidity or mortality. There is limited information on the incidence of bleeding following nasotracheal intubation reported in the literature. Tintinalli and Claffey^19^ observed 71 nasotracheal intubations carried out by inexperienced practitioners, under supervision, in an emergency setting. They demonstrated a hemorrhage rate of 17%. However, in only one of these patients was the hemorrhage rated as severe, defined as gross blood suctioned from the mouth or requiring nasal packing. On the other hand, bleeding from instrumentation of the upper airway via tracheal intubation has been shown to be associated with bacteremia.^20^^,^^21^ Several authors^22-29^ reported that bacteremia occurs more frequently after nasotracheal than orotracheal intubation.

***Limitations: ***One major limitation in this study was the inclusion criteria for patients. Only patients older than 4 years scheduled for nasotracheal intubation were included. For ethical reasons, Children under 4 years of age are intubated only orally in routine dental practice under general anesthesia in our dental hospital clinic. Thus, younger children who received DRGA involving orotracheal intubation were excluded from the present study.

## CONCLUSION

Based on this study’s results, the following conclusions can be made:

1. The most common experienced postoperative symptom after DRGA was bleeding.
2. Postoperative discomfort was related to number of the extractions. Children who had 4 or more extractions were more likely to experience pain.
3. Nasal bleeding was an uncommon complication and did not cause serious morbidity or mortality in children intubated nasotracheally.
4. Although an antiemetic drug (Metoclopramide) was administered to patients in the preoperative period, nausea and vomiting was observed in both the nasal intubation and oral intubation groups at a level that could not be ignored.
